# Congenital absence of superficial posterior compartment calf muscles

**DOI:** 10.1007/s10195-013-0256-9

**Published:** 2013-08-08

**Authors:** Saket Tibrewal, Faisal Alyas, Krishna Vemulapalli

**Affiliations:** 1 Queen’s Hospital, Romford, UK; 2 59 Elmstead Lane, Chislehurst, Kent BR7 5EQ UK

**Keywords:** Congenital, Muscle agenesis, Absence

## Abstract

Although various congenital abnormalities have been described, congenital absence of calf musculature is extremely rare, with only one report on its complete absence. We are the first to describe a case of congenital absence of muscles of the superficial posterior compartment of the calf presenting in a toddler. The child presented with a history of a painless limp, however no significant difference was found in functional gait analysis. We suggest that such cases should be monitored and parents can be reassured that no immediate treatment is required.

## Introduction

The incidence of congenital absence of muscles is rare and reported to be 1:11,000 [1]. Although various congenital muscular abnormalities have been described, there is only one report on the complete absence of calf musculature [2]; that case was diagnosed in the early postnatal period. We are the first to describe a case of congenital absence of muscles of the superficial posterior compartment of the calf presenting in a toddler.

## Case report

A 2-year, 4-month-old Afro-Caribbean boy presented to our clinic with a history of abnormal gait and a size discrepancy in the legs noticed by the mother. He was born at term by normal spontaneous vaginal delivery following an uneventful pregnancy. No abnormalities were noted at birth, and he had achieved normal developmental milestones. From the age of 6 months, the patient’s mother noticed that his left leg appeared different to the right; as he grew, it appeared that the right leg grew faster than the left. This difference became more apparent when the child started walking, and the mother noticed the gradual onset of a painless limp and leaning towards the left side. She also noticed a difference in the size of the feet when the child started using footwear, with the left shoe always being too big for the left foot. At this stage, the mother noticed that the child was occasionally tripping when walking or running and that the lower left leg was much thinner than the right.

On review in our clinic, the patient was walking and running appropriately. There were no tripping, functional nor balance difficulties. Leg lengths, foot lengths and thigh circumferences were equal; however, there was an obvious asymmetry of the calves, with the left smaller than the right. We found a 4-cm difference in calf circumference measured at a fixed point (4 cm) below the lower pole of the patella. Neurological, vascular and cutaneous examination of the lower limbs was normal. There were no abnormalities of upper limbs or spine.

**Fig. 1:**
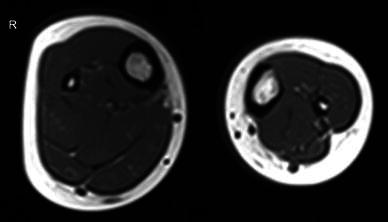
Axial T1-weighted spin-echo magnetic resonance image (TE-11 TR-656) sequences through the proximal aspect of both lower limbs. This demonstrates a normal superficial posterior compartment on the right. There is absence of most of the superficial compartment on the left, apart from atrophied lateral gastrocnemius and compensatory hypertrophy of the left deep posterior compartment and peroneal compartment

**Fig. 2:**
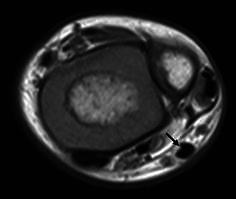
Axial T1-weighted spin-echo magnetic resonance image (TE-10, TR-442) sequence of the left leg just above the ankle. *Arrow* identifies atrophied Achilles tendon

**Fig. 3:**
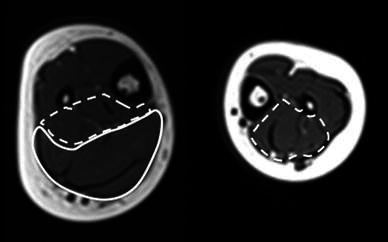
Magnetic resonance imaging scan of both limbs showing absent musculature in the left. Axial T1-weighted spin-echo (TE-11 TR-656) sequences through the proximal aspect of both lower limbs demonstrating the absent superficial posterior compartment on the *left*. Note the compensatory hypertrophy of the deep compartment and peroneal compartment muscles on the *left*. The *solid line* represents the superficial posterior compartment. The *interrupted line* represents the deep posterior compartment

**Fig. 4:**
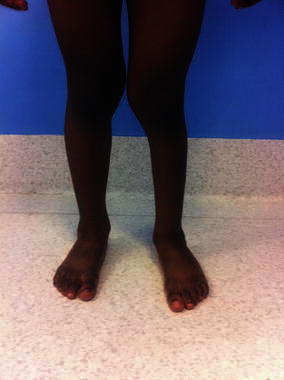
Difference in calf size from the front

**Fig. 5:**
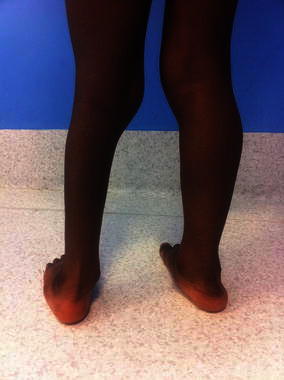
Difference in calf size from behind

Radiographs of the lower limbs revealed normal bony architecture; renal ultrasound was normal, as was magnetic resonance imaging (MRI) of his brain and spine. MRI of his lower limbs (Figs. 1, 2, 3) confirmed complete absence of the soleus and medial gastrocnemius, with a rudimentary lateral gastrocnemius arising from the semimembranosus to form the Achilles tendon. The MRI also revealed hypertrophy of the deep posterior compartment and peroneal compartment muscles on the left side. Creatine kinase was mildly raised at 193 IU/L (normal 0–170 IU/L), but all other blood markers and genetic testings were normal. Static and dynamic gait analysis revealed no significant difference between feet, including foot phasing, calcaneal stance angles and rearfoot/forefoot angles (Table 1). We counseled the parents and reassured them that no intervention was required. At latest follow-up, the child was 5 years and 4 months old and progressing well clinically. Although there remains an obvious difference in calf size (Figs. 4, 5), there are no functional difficulties. The patient’s parents gave informed consent prior to our writing this report.

**Table 1:** Comparison of gait analysis findings

	Left foot	Right foot
Rearfoot phase (%)	6.9	9.09
Midfoot phase (%)	41.36	43
Forefoot phase (%)	55	50
Neutral calcaneal stance angle	6° varus	5° varus
Relaxed calcaneal stance angle	11° valgus	10° valgus

## Discussion

Congenital absence of muscles is rare, with those of the axial skeleton being the most frequently absent. Within this group, the most commonly described are pectoralis major and trapezius [3, 4]. The appendicular skeleton, however, is even more rarely affected. Reported absences include thigh muscles [4], gluteal muscles [5], muscles of the anterior compartment of the leg [6] and elbow flexors [7]. In our case, there was absence of the musculature of the superficial posterior compartment of the calf, which only became clinically apparent once the child began walking. The only similar case described an absence of the entire (superficial and deep) posterior compartment musculature in addition to the Achilles tendon. This case was first identified in the immediate postnatal period and thought to be a calcaneus deformity of the foot. In our case, an Achilles tendon was present, and no abnormality was discovered until the child started ambulating.

The aetiology of this form of anomaly can be divided into vascular, mechanical, teratogenic, congenital or developmental [2]. In our case, the origin was congenital: there was no history of a vascular injury, other insult or trauma; brain and spinal imaging were normal; there were no visceral or skeletal deformities, no clinical evidence of neuromuscular disorders and no genitourinary abnormalities. Other than a cosmetic deformity, no functional deficit was apparent. This was initially confirmed by gait analysis, and no treatment was required. Even though this patient has no soleus or medial head of the gastrocnemius, there is a rudimentary lateral head of the gastrocnemius, with an atrophied Achilles tendon (Fig. 1). MRI also revealed hypertrophy of the deep posterior compartment muscles and peroneal compartment muscles on the left side. We believe the patient is able to plantar flex his ankle using these rudimentary superficial compartment muscles and the compensatory hypertrophied deep compartment muscles (Figs. 3, 4, 5).

We suggest there is potential for future problems with the Achilles mechanism and plantarflexion in this patient. We counseled the family with regards to this possibility and suggested that, although we do not expect any difficulties, the child be monitored locally until he has fully developed to ensure no such problems arise. Congenital absence of calf musculature is extremely rare. We suggest that similar patients should be monitored as they develop and that parents can be reassured that no immediate treatment is required.

## References

[1] Kocak G, Aysun S, Akhan O (1995). Unilateral agenesis of the sternocleidomastoid muscle. Turkish J Paediatr.

[2] Flynn JM, Ramirez N, Cornier AS, Colon-Negron E (2007). Unilateral congenital absence of the calf muscle. J Pediatr Orthop B.

[3] Bing R (1902). Ueber angeborence muskeldefecte. Virchows Arch.

[4] Petersen JE, Currarino G (1981). Unilateral absence of thigh muscles confirmed by CT scan. Paediatr Radiol.

[5] Carnevale A, Del Castilllo V, Sotillo AG, Larrondo J (1976). Congenital absence of gluteal muscles. Report of two sibs. Clin Genet.

[6] Ohno K, Monji J, Sasaki T, Inoue K, Shiono H (1986). Congenital absence of anterior compartment muscle of both legs: a case report. Nippon Seikeigeka Gakkai Zasshi.

[7] Netscher DT, Aliu O, Samra S, Lewis E (2008). A case of congenital bilateral absence of elbow flexor muscles: review of differential diagnosis and treatment. Hand (NY).

